# Lungs in Heart Failure

**DOI:** 10.1155/2012/952741

**Published:** 2012-12-24

**Authors:** Anna Apostolo, Giuliano Giusti, Paola Gargiulo, Maurizio Bussotti, Piergiuseppe Agostoni

**Affiliations:** ^1^Centro Cardiologico Monzino, IRCCS, 20138 Milan, Italy; ^2^Dipartimento di Medicina Clinica, Scienze Cardiovascolari ed Immunologiche, Università degli Studi di Napoli, 80131 Napoli, Italy; ^3^Divisione di Cardiologia Riabilitativa, Fondazione Salvatore Maugeri, IRCCS, 20138 Milan, Italy; ^4^Dipartimento di Scienze Cliniche e di Comunità, Università di Milano, 20138 Milan, Italy; ^5^Division of Pulmonary and Critical Care and Medicine, Department of Medicine, University of Washington, Seattle, WA 98195-6420, USA

## Abstract

Lung function abnormalities both at rest and during exercise are frequently observed in patients with chronic heart failure, also in the absence of respiratory disease. Alterations of respiratory mechanics and of gas exchange capacity are strictly related to heart failure. Severe heart failure patients often show a restrictive respiratory pattern, secondary to heart enlargement and increased lung fluids, and impairment of alveolar-capillary gas diffusion, mainly due to an increased resistance to molecular diffusion across the alveolar capillary membrane. Reduced gas diffusion contributes to exercise intolerance and to a worse prognosis. Cardiopulmonary exercise test is considered the “gold standard” when studying the cardiovascular, pulmonary, and metabolic adaptations to exercise in cardiac patients. During exercise, hyperventilation and consequent reduction of ventilation efficiency are often observed in heart failure patients, resulting in an increased slope of ventilation/carbon dioxide (VE/VCO_2_) relationship. Ventilatory efficiency is as strong prognostic and an important stratification marker. This paper describes the pulmonary abnormalities at rest and during exercise in the patients with heart failure, highlighting the principal diagnostic tools for evaluation of lungs function, the possible pharmacological interventions, and the parameters that could be useful in prognostic assessment of heart failure patients.

## 1. Introduction

Not only heart is involved in chronic heart failure but also lung, kidney, peripheral and respiratory muscles, chemo-ergoreceptors, neurohormonal mechanisms, mitochondria, all play a major role in determining the complex clinical syndrome of chronic heart failure. Indeed, energy deficit is a relevant contributor to the development of cardiac and skeletal myopathy. In heart failure several functions of muscle bioenergetics are altered such as oxygen availability, substrate oxidation, ATP production by the mitochondria, and transfer to contractile apparatus [1]. Notably, the clinical syndrome of heart failure is characterized by symptoms apparently unrelated or partially related to the heart, such as fatigue, dyspnea, anxiety, and exercise intolerance.

Dyspnea, either at rest or during exercise, is one of the main symptoms in heart failure. Indeed, the most often used heart failure grading methodology, the NYHA classification, is based on dyspnea. Dyspnea is the result of a neurological reconstruction of an abnormal physiological condition characterized by hyperventilation and by high ventilation to metabolic demand ratio. This leads to a reduced ventilatory efficiency, which is physiologically defined as the amount of ventilation needed to eliminate a given amount of CO_2_. The excess of ventilation is due to an increase of dead space/tidal volume (VD/VT) ventilation and of ventilatory drive from peripheral chemo- and ergoreceptors. The reduced ventilatory efficiency during exercise is used more and more as a prognostic marker in heart failure. In addition, an improvement of the efficiency of ventilation is among the goals of heart failure therapy. The following review will examine the role of different mechanisms underlying ventilatory efficiency evaluated with spirometry, lung gas diffusion measurement, and cardiopulmonary exercise test in heart failure patients. 

## 2. Lung Abnormalities at Rest

Pulmonary abnormalities are part of the heart failure syndrome, as both lung mechanics and alveolar-capillary gas exchange are impaired [2–5]. In heart failure, pulmonary abnormalities may be due to respiratory comorbidities but also to heart failure itself. Therefore, standard spirometry and resting lung diffusion for carbon monoxide (DLCO) [6] provide an integrated evaluation of the respiratory function and should be performed in all heart failure patients. Moreover, it may be useful to split DLCO into its two subcomponents either using the classic Roughton and Forster method or nitric oxide lung diffusion. Accordingly, Dm, the resistance to molecular diffusion of carbon monoxide across the alveolar-capillary membrane, and Vcap, the resistance to carbon monoxide binding to hemoglobin, the so-called pulmonary capillary blood volume, can be calculated [7]. 

### 2.1. Spirometry

Spirometry is the preliminary pulmonary function test to assess respiratory mechanics.

**Figure 1:**
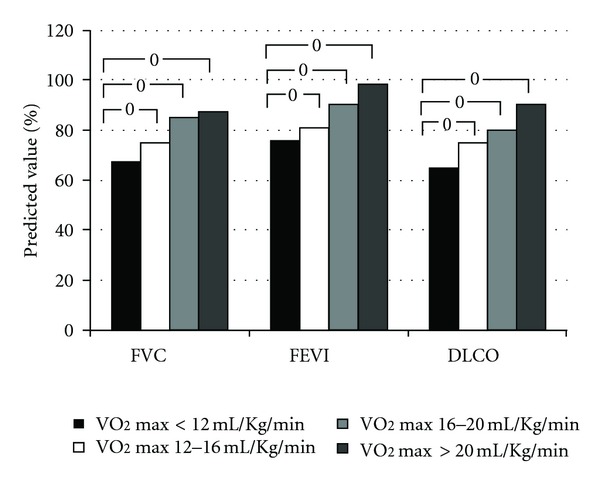
Lung function at rest in 190 heart failure patients in stable clinical condition. Patients were grouped according to exercise capacity. From left to right: peak VO_2_ < 12 mL/min/kg (black bars), peak VO_2_ = 12–16 mL/min/kg (white bars), peak VO_2_ = 16–20 mL/min/kg (grey bars), and peak VO_2_ > 20 mL/min/kg (dashed bars). FVC: forced vital capacity; FEV1: forced expiratory volume 1 second; DLCO: lung diffusion for carbon monoxide. Data from [8, Table 1].

Wasserman et al. [5] showed in a large multicenter study that, at rest, forced expiratory volume in the first second and vital capacity are either normal or proportionately reduced in heart failure. More recently, Agostoni et al. [8] demonstrated that pulmonary function at rest is usually normal in patients with moderate heart failure, while a restrictive lung disease is observed in 50% of patients with severe heart failure (Figure 1). Cardiac enlargement in heart failure appears to be involved in causing restrictive lung pattern [4, 9]. Indeed, a negative correlation between cardiac size, as analyzed by the cardiothoracic index at chest X-ray, and lung function parameters, including alveolar volume, has been described [4]. Notably, Palermo et al. [10] showed that, in heart failure, pulmonary function varies in relationship with the position of the body, being worst in the lateral decubitus. The larger the heart, the greater the difference in lung function between the sitting position and the lateral decubitus. Moreover, McCormack [11] showed that the restrictive lung disease secondary to severe heart failure seems to be completely reversible after cardiac transplantation, with an increase in forced vital capacity after transplantation, directly correlated with the decrease in cardiac volume.

### 2.2. Lung Diffusion

Alterations of respiratory mechanics and of gas exchange capacity are strictly related in heart failure patients. Reduction in the pulmonary DLCO is well documented in heart failure [3, 13–15]. The acute pathogenetic mechanisms of lung diffusion abnormalities in heart failure are related to interstitial edema, alveolar-capillary membrane hydrostatic injury, and altered alveolar fluid clearance. These mechanisms result in a remodeling process that causes a persistent reduction in alveolar-capillary membrane conductance and lung diffusion capacity. Indeed, Mettauer et al. [16] demonstrated that DLCO is only partially restored after cardiac transplantation. DLCO improves less in patients with long standing heart failure. This implies that in heart failure the alveolar-capillary membrane undergoes changes that are only partially reversible with heart failure treatment [17]. 

Reduced Dm is the main component of impaired pulmonary gas transfer in heart failure [18]. Puri et al. [18] demonstrated that Dm decreases and Vcap increases in relationship with the severity of the disease. The Vcap increase was interpreted as a compensatory mechanism aimed, by pulmonary vessel recruitment, at preserving alveolar-capillary diffusion. Vcap tends to increase in patients with stable heart failure, but could decrease in the advanced stages [19]. Agostoni et al. [8] showed that in severe heart failure in stable clinical condition there are few alveolar-capillary units at work (low alveolar volume), characterized by greater efficiency (high Dm/Vcap ratio) compared to alveolar-capillary units in intermediate chronic heart failure severity. However, the physiological mechanism behind this phenomenon is still undefined. Indeed, Vcap is related to the amount of hemoglobin participating in gas exchange, which, on its turn, depends from hemoconcentration, cardiac output, and the amount of capillary vessels in the ventilated airways. The latter is related to pulmonary venous pressure. 

In heart failure, the alveolar-capillary membrane surface area available for gas exchange is reduced and partially responsible for low Dm. Furthermore, Dm reduction remains even after correction for alveolar volume (Dm/VA) [18]. In fact, Dm is affected by several factors including interstitial edema that increases the distance between alveolar gas and red blood cells, fibrosis, inability to further activate the pump mechanism at the alveolar surface which enhances chlorine and sodium transport [20], and peribronchial edema that may reduce ventilation to some lung units. Moreover, there is a strict correlation between hemodynamics and Dm, as proven by the observation that Dm decreases after infusion of a small amount of saline (150 mL) [21] or after exercise [22, 23].

Nevertheless, we must underline that, albeit several heart failure models have been prepared to study the correlation between increased congestion of lung interstitial space and pulmonary function, a reliable model of lung function abnormalities mimicking those present in heart failure does not exist. Studies in healthy humans [24–26] demonstrated a reduced vital capacity, forced expiratory volume, and total lung capacity with a preserved DLCO after a rapid increase fluid content. This observation is consistent with some clinical findings. For instance, ultrafiltration, which acutely reduces the congestion of lung interstitium, improves lung mechanics but not DLCO in heart failure [17, 27].

DLCO is a limiting factor for exercise performance [28, 29], which improves (below sea level, as in the Dead Sea) or worsens at different altitudes more in heart failure patients with reduced DLCO compared to subjects with a normal DLCO [30, 31]. DLCO and Dm abnormalities have a relevant prognostic capacity in heart failure [32], although we do not know whether an improvement of DLCO with treatment is associated with an improvement of prognosis. 

More recently, a new biomarker of alveolar-capillary membrane damage has been described [33, 34]. Increased circulating plasma levels of surfactant protein B have been reported in heart failure patients with a good correlation with heart failure severity. The mature form of surfactant protein B plays a crucial role in the formation and stabilization of pulmonary surfactant film. The level of surfactant protein B correlates with lung diffusion as well as peak VO_2_ and ventilation versus CO_2_ production (VE/VCO_2_) slope [34]. The clinical applicability of this biomarker is unclear at present.

## 3. Lung Abnormalities during Exercise

Cardiopulmonary exercise test (CPET) with incremental increases in workload is considered as the “gold standard” when studying the cardiovascular, pulmonary, and metabolic adaptations to exercise in cardiac patients.

Traditionally, ventilatory limitation to exercise is assessed by measuring the breathing reserve calculated as the difference between minute ventilation at peak exercise and maximal voluntary ventilation or some estimate of the maximal voluntary ventilation (typically the forced expiratory volume in the first second multiplied by 35 or 40). Any difference >15 L/min or breathing reserve >20–40% is interpreted as consistent with exercise not being limited by ventilation [35]. In normal individuals, there is a progressive increase of ventilation (VE) during exercise, due to both VT and respiratory rate increase. The increase in VT mainly occurs at the beginning of exercise, whereas respiratory rate typically increases more toward peak exercise.

**Figure 2:**
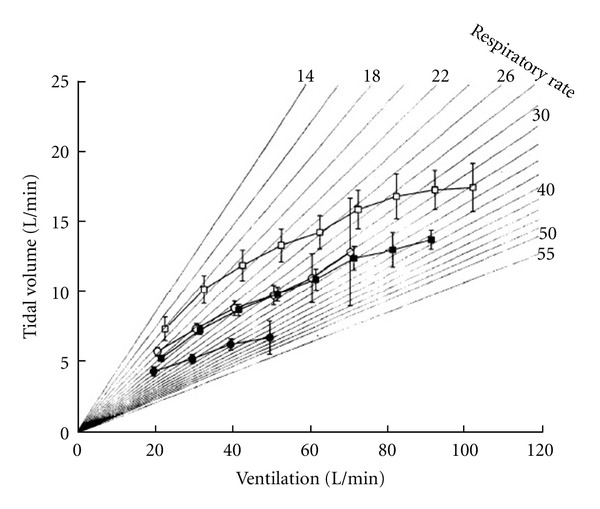
Tidal volume versus ventilation in patients with severe heart failure (peak VO_2_ < 12 mL/min/kg, black circles), moderate to severe heart failure (peak VO_2_ 12–16 mL/min/kg, black squares), moderate (peak VO_2_ > 16 mL/min/kg, empty circles), and normal subjects (empty squares). From [5]. Reproduced with permission.

CPET reveals an increased VE, at comparable levels of effort, in heart failure patients with respect to age-matched normal individuals [36]. At a given work rate, heart failure patients show a higher VE than normal subjects, the result of an exaggerated respiratory rate response, and a truncated VT response [5] (Figure 2).

Ventilatory response may be abnormal during exercise despite normal breathing reserve as showed in different settings and in heart failure patients [12, 37, 38]. Lung hyperinflation and expiratory flow limitation can cause fatigue of the inspiratory muscles; therefore, it becomes clear that breathing reserve is insufficient for a comprehensive and precise assessment of the contribution of the respiratory system to physical exercise in physiologic and disease conditions. Indeed, diminished respiratory muscle strength has been demonstrated in heart failure patients. Mouth inspiratory and expiratory pressures are reduced in heart failure compared to normal subjects, and they seem to be correlated with exercise capacity [39]. Moreover, inspiratory muscle strength has independent prognostic value in heart failure. The results of trials with inspiratory muscle training [40] indicate that this intervention improves exercise capacity and quality of life in heart failure. Some benefit from muscle training may be accounted for by the attenuation of the inspiratory muscle metaboreflex. Furthermore, inspiratory muscle training results in improved cardiovascular responses to exercise. These findings suggest that routine screening for intercostal muscle weakness is advisable in patients with heart failure and specific inspiratory muscle training and/or aerobic training are of practical value in the management of these patients.

**Figure 3:**
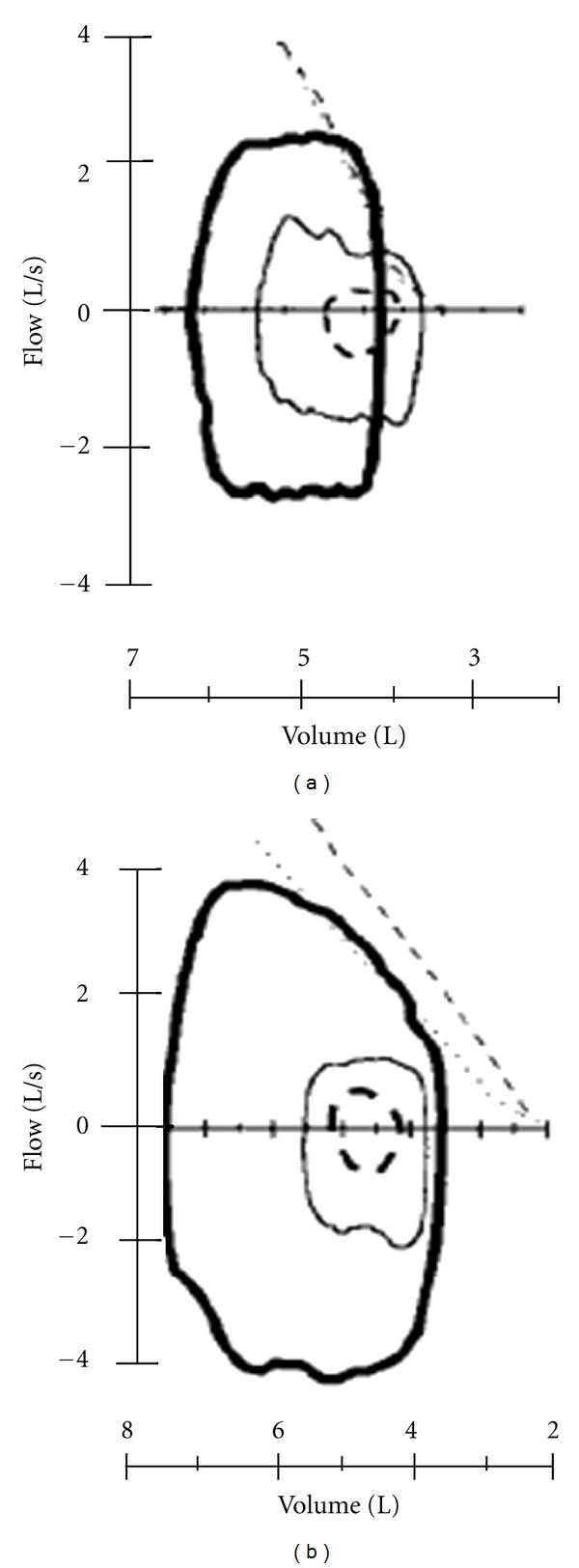
Tidal flow-volume loops at rest (dashed lines) at 40% of maximal ventilation (thin solid lines), and at maximum exercise (thick solid lines) in typical heart failure (a) and normal (b) subjects. The 2 oblique lines on flow-volume loops are partial forced expiratory flows recorded at rest (dotted line) and at maximum exercise (dashed line). From [12].

More detailed information about ventilatory abnormalities during exercise in heart failure is provided by analysis of the spontaneous expiratory flow-volume loop relative to the maximal forced curve. Expiratory flow limitation is reached when the right upper corner of the flow-volume loop curve is very close to the maximal flow volume curve registered at rest. When this happens, subjects stop the effort or proceed through the exercise but the flow volume curve shifts to the left, away from the functional residual capacity, in a region where the cost of breathing increases. Johnson et al. [41] and Agostoni et al. [12] showed that in heart failure, because of lung stiffness, the spontaneous flow-volume loop reaches the maximal flow-volume loop and the expiratory flow reserve is dramatically reduced, requiring an increase in end-inspiratory lung volume. An increase in end-inspiratory lung volume raises work of breathing and decreases inspiratory endurance time [41]. In addition, after exercise in healthy subjects, the maximal flow-volume loop is increased due a bronchodilation induced by exercise; this is not the case in heart failure [42].  Figure 3 describes an example of flow-volume curves' behavior during exercise in a normal subject and in a heart failure patient. Moreover, Bussotti et al. [43] showed that maximal flow-volume loops maneuver does not interfere with the main functional parameters used for the interpretation of CPET. Consequently, with a single CPET both flow-volume curve and ventilation efficiency (VE/VCO_2_ slope) can be evaluated.

### 3.1. Ventilation versus VCO_2_

**Figure 4:**
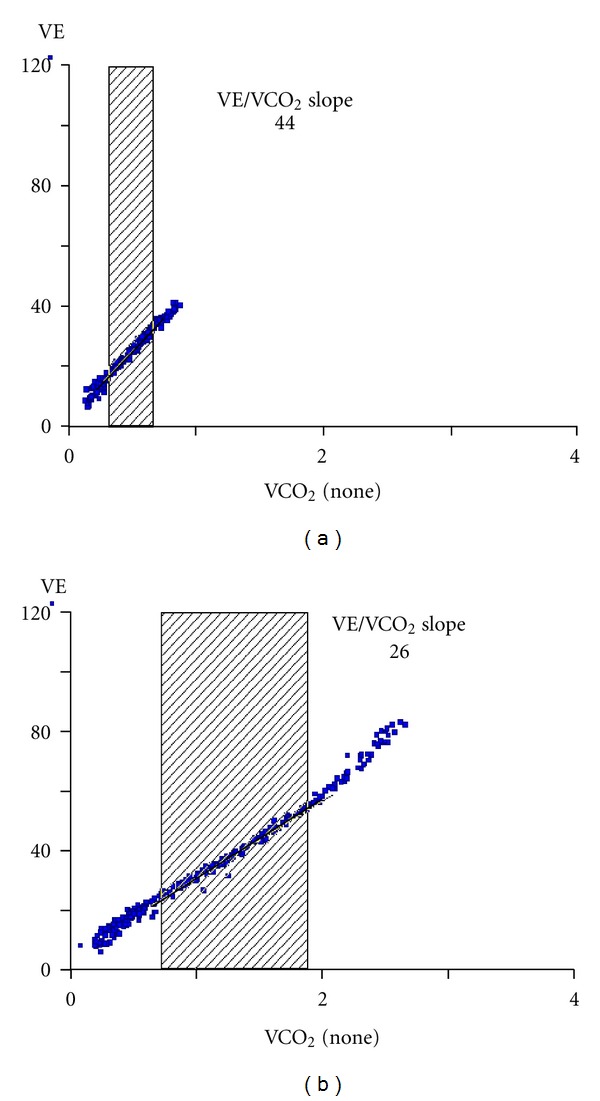
VE/VCO_2_ slope in an heart failure patient (a) and an healthy control (b).

In normal subjects the relation of VE versus VCO_2_ is characterized during CPET progressive work load increase by three linear relationships. The three slopes are progressively steeped. This is due to the change in the functional parameters governing the VE versus VCO_2_ relationship and specifically VO_2_, VCO_2_, and pH [44]. Clinically the VE versus VCO_2_ slope is measured from the first minute after the beginning of loaded pedaling to the end of isocapnic period (Figure 4), albeit some authors [45] suggest to measure the slope obtained considering the entire exercise as a single linear relationship. Differently the VE/VCO_2_ ratio declines at the beginning of exercise, reaches a plateau, and increases in the last part of exercise, when metabolic acidosis becomes unbuffered. In heart failure exercise is characterized by hyperventilation, which is likely due to several causes, including alteration of lung mechanics, reduced lung diffusion, increased CO_2_ production due to early lactic acidosis, increased VD, decreased ventilatory efficiency, and overactive reflexes from metaboreceptors, baroreceptors, and chemoreceptors. In other words, in heart failure patients, besides abnormalities in the lung, either due to mechanics or to gas exchange, also ventilatory control is altered. The latter is likely a part of deranged cardiorespiratory reflex control. Indeed, a direct link between exercise hyperventilation and impaired reflexes which control heart rate and blood pressure has been convincingly proposed [46] providing the physiological base of the link between hyperventilation and poor prognosis in heart failure. Ventilatory efficiency is best defined by the relationship of the amount of ventilation required to eliminate a given amount of CO_2_. Efficiency of ventilation can be expressed as a slope of the VE/VCO_2_ relationship or as VE/VCO_2_ ratio measured either at the anaerobic threshold or as the lowest value recorded during exercise. The modified alveolar equation [44] concisely describes the determinants of the steepness which VE rises with respect to VCO_2_:

(1) $$\begin{array}{c} \frac{\text{VE}}{\text{V}{\text{CO}}_{2}}=\frac{K}{\left[\text{Pa}{\text{CO}}_{2}\times \left(1-\text{VD}/\text{VT}\right)\right]\;}, \end{array}$$

where [*K*/PaCO_2_ × (1 − VD/VT)] is the slope, *K* is constant to adjust for standard temperature pressure dry and body temperature pressure saturated and to convert fractional concentrations to pressures, PaCO_2_ is partial pressure of CO_2_ in arterial blood, and VD/VT is the fraction of VT that goes to VD. This equation is linear over a wide range of exercise. Figure 4 describes a theoretical example of VE/VCO_2_ slope in a normal subject and in a patient with heart failure. If PaCO_2_ is driven down by a high ventilatory drive from peripheral chemoreceptors or by ergoreceptors in skeletal muscles, the slope of the VE/VCO_2_ will increase as well as if VD/VT is high. Little is known about chemosensitivity in heart failure. It has been documented that central hypercapnic chemo-sensitivity is enhanced in patients with heart failure with central sleep apnea [47]. Chua et al. [48] demonstrated that there is increased hypoxic and central hypercapnic chemo-sensitivity in patients with heart failure, and that its suppression with dihydrocodeine is associated with a reduction of exercise ventilation, an improvement in exercise tolerance, and a decrease in breathlessness [49]. Piepoli et al. [50] showed that muscle reflex (ergoreflex) has an important effect on the ventilatory responses to exercise in heart failure compared to control subjects, and that training may reduce this exaggerated ergoreflex activity, thereby improving the response to exercise. The increase in reflex sensitivity may serve as a compensatory mechanism producing an increase in ventilatory response during exercise and thereby preserving blood gas homeostasis, also maintaining arterial oxygen concentration. Studies analyzing the effect of drugs interfering with chemo-sensitivity are on-going, and no data are available at present.

In normal subjects, VE/VCO_2_ relationship, for incremental exercise, is normally linear up to the “respiratory compensation point,” when ventilatory compensation begins in response to metabolic (lactic) acidosis. Over the linear phase of the VE/VCO_2_ relationship, below the respiratory compensation point, the profile of VE/VCO_2_ closely reflects that of VD/VT, providing information on ventilatory efficiency. In heart failure, hyperventilation is associated with an increased VD/VT and VCO_2_ and a lower PaCO_2_ when compared to similar normal subjects at a similar percent of VO_2_ peak [5]. Patients with heart failure often have a reduced tidal volume at heavy exercise [5], which would increase the VD/VT ratio; however, Buller and Poole-Wilson [51] showed that the increased ventilatory response to exercise in patients with heart failure is largely caused by mechanisms other than increased ventilation of anatomical VD [51]. In heart failure patients, in the absence of coexisting lung disease, this pattern of a high VD/VT ratio with normal arterial blood gases suggests that nonuniformity of ventilation/perfusion (V/Q) ratios in the lung is more likely caused by increased non-uniformity of perfusion than of ventilation [52]. A totally different behavior of the VE/VCO_2_ relationship is likely when respiratory comorbidities, such as emphysema, are present [53, 54].

The normal VE/VCO_2_ at the nadir is between 25 and 35. Normal values for these ventilatory equivalents with an end-tidal of CO_2_ (PetCO_2_) of approximately 40 mmHg suggest a normal VD/VT and uniform matching of V/Q [44].

In recent years, the VE/VCO_2_ slope has gained notoriety in the heart failure population as an outstanding prognostic marker. Several studies report the VE/VCO_2_ slope to be prognostically superior to peak VO_2_ to predict mortality [55–60]. Arena et al. [55] built a classificatory system based on exercise VE/VCO_2_ slope to stratify the risk of major cardiovascular events in heart failure. Corrà et al. [56] used VE/VCO_2_ slope (with a cutoff >34) for efficient prognostic stratification in patients with moderate to severe heart failure (defined as VO_2_/kg peak 10–18 mL/kg/min). Ponikowski et al. [60] showed that, even in heart failure patients with normal exercise performance and peak VO_2_ >18 mL/kg/min, abnormal exercise ventilation significantly discriminates survival. Guazzi et al. [61] suggested that the VE/VCO_2_ slope has a remarkable value for risk stratification even in patients with diastolic heart failure. 

Another way to assess ventilation during exercise is to analyze the relationship between end-tidal CO_2_ pressure (PetCO_2_) and VE during the isocapnic buffering period [44, 62]. At sea level, during isocapnic buffering period, there is a straight relationship between CO_2_ set point and ventilation. PetCO_2_, equivalent to PaCO_2_, during the isocapnic buffering period, is around 40 mmHg in normal subjects, higher in athletes [63] and lower in heart failure patients. Guazzi et al. [64] suggested that a low peak PaCO_2_ (and consequently a low peak PetCO_2_ and an elevated VD/VT) is a strong independent predictor of mortality in stable heart failure, and that a low peak PaCO_2_ is the most significant determinant of the prognostic value of a steep VE/VCO_2_ slope. Another ventilation-related index with a prognostic value in heart failure is Oxygen Uptake Efficiency Slope (OUES). OUES is derived from the relation between oxygen uptake (VO_2_ L/min) and VE (L/min) during incremental exercise. OUES is determined by the linear relation of VO_2_ (*y*-axis) versus the logarithm of VE (*x*-axis) during exercise [65].

The following equation was used to determine the relation between VO_2_ and VE:

(2) $$\begin{array}{c} \text{V}{\text{O}}_{2}=a\;\log\text{VE}+b.\; \end{array}$$

The differential of this equation by VE yields is

(3) $$\begin{array}{c} \frac{d\text{V}{\text{O}}_{2}}{d\text{VE}}=\frac{a\left(1/\log10\right)}{\text{VE}}, \end{array}$$

where *a* is the constant that represents the rate of increase in VO_2_ in response to VE. We define the constant “*a*” as the OUES. Baba et al. [65] emphasized the value of OUES as a submaximal, effort-independent, and objective parameter to estimate cardiorespiratory functional reserve, and they reported that OUES strongly correlates with VO_2_max⁡(*r* = 0.941).

Myers et al. [66], when defining a CPET score for predicting outcomes in heart failure, considered OUES to be a stronger predictor of risk than peak VO_2_. Davies et al. [67] claimed OUES as a prognostic marker in heart failure patients. Sun et al. [68] have recently described the Oxygen Uptake Efficiency highest Plateau (OUEP, i.e., oxygen uptake/ventilation = VO_2_/VE) as the best single predictor of early death (six months), in a cohort of 508 patients with low ejection fraction (>35%), with an Odds ratio for mortality of 13. When OUEP is combined with periodic breathing, the Odds ratio increases to 56.

### 3.2. Periodic Breathing

Periodic breathing is a ventilatory pattern, which is present in some heart failure patients both at rest and during exercise. Exercise-induced periodic breathing has been defined by Kremser et al. [69] as the presence of cyclic fluctuation of VE lasting longer than 66% of the exercise, with an amplitude of more than 15% of the average value at rest. It can be observed during the entire exercise or it disappears after a few minutes. The origin of periodic breathing is still unclear and several mechanisms have been proposed, mainly grouped into ventilatory (instability in the feedback ventilatory system) and hemodynamic (pulmonary blood flow fluctuations). Agostoni et al. [70], showed that, adding 250 and 500 mL of dead space, respectively, periodic breathing disappears earlier during exercise and suggested that low tidal volume and carbon dioxide apnea threshold are important contributors to periodic breathing. Schmid et al. [71] supposed that periodic breathing in heart failure might potentiate the negative effects of low cardiac output and high ventilation on exercise performance, and they concluded that the presence of periodic breathing negatively influences the exercise performance of heart failure patients, likely because of an increased cost of breathing. Indeed, periodic breathing disappearance during exercise showed to be associated with a more efficient oxygen delivery in most cases. Regardless, periodic breathing is associated with a worse prognosis [64, 72] in heart failure and reflects disease severity [73].

## 4. Therapeutic Interventions

Several reports showed that the respiratory system can be one of the targets for proper heart failure treatment. On this regard, several therapeutic interventions affect the ventilatory abnormalities both at rest and during exercise in heart failure patients. Indeed, ACE-inhibition improves pulmonary diffusion in heart failure [74]. ACE-inhibition could improve pulmonary hemodynamic, remove interstitial fluid and pulmonary vasoconstriction, and improve DLCO. The effect of ACE-inhibitors is counteracted by aspirin, suggesting that bradykinin metabolism has a role, and bradykinin is likely to participate to abnormal alveolar capillary gas diffusion regulation in heart failure. Most importantly the studies on ACE-inhibitors, with and without aspirin, showed a direct effect of these drugs on lung gas diffusion, in the absence of a hemodynamic effect [74]. Agostoni et al. [75] showed that spironolactone improves gas diffusion through the lungs in stable heart failure patients with impaired DLCO, possibly through a reduction of pulmonary fibrosis.

Also Beta-blockers affect DLCO diffusion. Beta-receptors in the lung are located on the alveoli, mainly Beta2-receptors, and on the airways (mainly Beta1-receptor). Carvedilol reduces DLCO, due to a reduction of membrane diffusion [62], while Bisoprolol does not modify DLCO [76]. Carvedilol and Bisoprolol have a different pharmacological action, blocking both Beta1- and Beta2-receptors (Carvedilol) or selectively Beta1-receptor (Bisoprolol). The pharmacological action of Beta-blockers can explain the different actions on DLCO diffusion. Carvedilol improves clinical conditions, without affecting exercise performance. Carvedilol, but not Bisoprolol, reduces hyperventilation through exercise [77], as shown by a lower VE/VCO_2_ slope, consequent to an increase in the arterial CO_2_ set point [62, 78]. The improvement of the clinical conditions of heart failure patients treated with Carvedilol could be associated with reduction of the inappropriately elevated ventilation levels observed during exercise and consequently dyspnea.

A direct effect of Carvedilol on chemoreceptors activity has been recently suggested [79]. This reduction of hyperventilation during exercise is present both in normoxia (equivalent to sea level) and in hypoxia (equivalent to 2,000 m altitude). The reduction of hyperventilation by Carvedilol has a negative influence on arterial PO_2_ during exercise at a simulated altitude of 2,000 m [78]. 

In conclusion, lung abnormalities have a major role in heart failure syndrome. Indeed, lung abnormalities influence the clinical setting being dyspnea, a frequently reported heart failure symptom, affecting exercise performance, providing a tool to grade heart failure severity and to predict its prognosis, and finally being the target of therapy with several drugs commonly used in heart failure.
